# Inverter Circuits Using ZnO Nanoparticle Based Thin-Film Transistors for Flexible Electronic Applications

**DOI:** 10.3390/nano6090154

**Published:** 2016-08-23

**Authors:** Fábio F. Vidor, Thorsten Meyers, Ulrich Hilleringmann

**Affiliations:** Sensor Technology Department, Paderborn University, 33098 Paderborn, NRW, Germany; meyers@sensorik.upb.de (T.M.); hilleringmann@ieee.org (U.H.)

**Keywords:** nanoparticles, ZnO, thin-film transistor, inverter circuit, low-temperature, low-cost, flexible electronics

## Abstract

Innovative systems exploring the flexibility and the transparency of modern semiconducting materials are being widely researched by the scientific community and by several companies. For a low-cost production and large surface area applications, thin-film transistors (TFTs) are the key elements driving the system currents. In order to maintain a cost efficient integration process, solution based materials are used as they show an outstanding tradeoff between cost and system complexity. In this paper, we discuss the integration process of ZnO nanoparticle TFTs using a high-*k* resin as gate dielectric. The performance in dependence on the transistor structure has been investigated, and inverted staggered setups depict an improved performance over the coplanar device increasing both the field-effect mobility and the *I*_ON_/*I*_OFF_ ratio. Aiming at the evaluation of the TFT characteristics for digital circuit applications, inverter circuits using a load TFT in the pull-up network and an active TFT in the pull-down network were integrated. The inverters show reasonable switching characteristics and *V*/*V* gains. Conjointly, the influence of the geometry ratio and the supply voltage on the devices have been analyzed. Moreover, as all integration steps are suitable to polymeric templates, the fabrication process is fully compatible to flexible substrates.

## 1. Introduction

Nowadays, innovative products exploring the flexibility and the transparency of modern semiconducting materials are reaching the market maturity. Driven by the internet of things (IoT), prototypes of flexible displays, of radio identification tags and of wearable electronic skins, for instance, are equipped with sensor arrays connecting different applications of distinct sectors, as energy, fashion and healthcare. Different scientific groups and companies are focused on the advancement of this technology, in which thin-film transistors (TFTs) are essential elements being responsible for driving the currents in the system [1,2,3]. The transistor geometry and the choice of the used materials have a crucial impact on the field of possible applications either for analog or digital circuits. Silicon based materials, for instance, were investigated as active semiconductor. However, the high temperatures required during their processing prevent the integration on flexible substrates. Organic semiconductors have also been researched intensively for more than 25 years [4]. Nevertheless, as degradation effects influence the electrical performance of the TFTs operating under ambient conditions, the lifetime of such systems is limited [5]. To overcome this challenge, on the one hand, passivation layers or encapsulations are used increasing the production cost. On the other hand, new synthetized thiophene derivates, such as Dinaphtho[2,3-*b*:2′,3′-*f*]thieno[3,2-*b*]thiophene (DNTT) and 2,7-dioctyl[1]benzothieno[3,2-*b*]benzothiophene (C_8_-BTBT), have shown better electrical stability to ambient air due to their larger ionization potential. Therefore, they are currently in the focus of different research groups [1,6,7]. 

Metal oxide compounds are another group of materials that have been actively researched for the low-cost sector and for large-area applications. Among these, zinc oxide based materials have shown outstanding electrical, chemical and sensory characteristics [8]. Moreover, ZnO is transparent to the visible light spectrum due to its direct band gap of about 3.3 eV at room temperature [9,10]. The integration of TFTs using ZnO as active semiconductor is widely spread in the literature [2]. Different deposition methods and compounds have been investigated depicting appropriate characteristics for diverse areas of interest. High performance metal oxide based TFTs were already reported. Nevertheless, they require the use of a certain amount of either rare or expensive elements in their composition, as indium or gallium, or the use of vacuum techniques, as sputtering or atomic layer deposition, during the integration process of the semiconductor. Concerning the low-cost sector, high throughput processes and cost efficient methods and materials have priority. The application of solution-based materials combined with integration techniques as spray coating, roll-to-roll, doctor blade or inkjet-printing have shown the possibility to fabricate systems on large surface area substrates. Even using cost efficient materials and methods, the integrated systems have been able to provide low-cost devices without drastically deterioration of the transistor performance. The use of precursors to achieve reliable active semiconducting layers commonly require a high temperature annealing step for the dehydroxylation reaction limiting the range of substrates and materials used in the system. Moreover, metal oxide precursors are commonly based on chlorine, nitrate or acetate solutions; therefore, beside the different temperature requirements, the chemical compatibility with the previously deposited materials must also be analyzed. 

The use of a nanoparticle dispersion of the semiconducting material avoids most of the issues related to temperature and chemical compatibility. In this manner, the fabrication of high quality nanocompounds is independent of the device integration, and only a low-temperature annealing process is required for the evaporation of the dispersant used in the nanoparticle solution. As the growth technology of ZnO nanoparticle has shown outstanding characteristics [9,10], nanostructures in different sizes and shapes, as well as dispersed in various solutions, such as water, ethanol and isopropanol, are available in the market. The choice of the nanoparticle dispersion can be adjusted depending on the requirement of the integration process. In contrast to the appreciated low annealing temperature processes the large surface area of the used nanoparticles increases the interaction with the ambient. This interaction is reported to induce instabilities to the electrical characteristics of the transistor if a passivation layer or a stabilization step is not applied [11,12,13].

**Figure 1:**
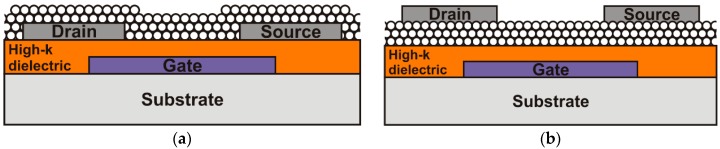
Schematic cross section of ZnO TFTs applying (**a**) inverted coplanar and (**b**) inverted staggered setups.

Another important aspect regarding the device fabrication is the transistor topology, which defines the integration procedure order. The two commonly used layouts to integrate either inorganic or organic based TFTs are the inverted coplanar (Figure 1a) and inverted staggered (Figure 1b) setups. The main difference between these structures is the order of the semiconducting layers deposition in relation to the integration of the drain and source electrodes. For the coplanar setup the semiconductor material will not be influenced by any additional integration step of the TFT. Conversely, for the staggered setup the semiconducting layer has to endure the integration process of the drain and source electrodes. This setup, nevertheless, is reported to have a better contact quality between the drain and source material and the active semiconductor [14]. In order to avoid the chemical and physical stress suffered by the semiconducting layer, several groups make use of shadow masks instead of conventional lithography technique. However, this method prevents the integration of high density circuits, limits the minimum transistor size to about 10 µm, and it is not entirely suitable for large area substrates. Other setups using top gates are also available, although due to the roughness at the semiconductor and the gate dielectric interface [15,16], a limited transistor performance is observed. Therefore, bottom gate structures (inverted setups) are preferable and largely used.

In this paper, we report the integration and electrical characterization of low-cost ZnO nanoparticle based TFTs for flexible electronic systems. As gate dielectric, a high-*k* nanocomposite combining the flexibility of polymeric materials and the high dielectric constant of inorganic compounds was used. Additionally, we discuss the performance of both inverted coplanar and staggered transistor setups. Moreover, inverter circuits are also presented in order to evaluate the TFTs for digital circuit applications. As all integration process steps are fully compatible to polymeric materials, as demonstrated in our previous work [17], the integration of the TFTs and inverter circuits on flexible substrates is feasible.

## 2. Results and Discussion

In this section, the characteristics of the TFTs regarding the properties and performance of the inverted coplanar and inverted staggered setups are analyzed. Moreover, inverter circuits availing the possibility to integrate cost-efficient circuits on flexible substrates are shown. In both setups, the active semiconductor is exposed to ambient air requiring a stabilization or an application of a passivation layer in order to avoid instabilities in the TFT operation. For this reason, prior to the analysis of the electrical characteristics of the ZnO nanoparticle TFT setups a discussion addressing the semiconducting film stabilization is presented. 

### 2.1. ZnO Stabilization Treatment

The interaction between molecules present in the atmosphere and metal oxides is known and utilized in gas sensor applications [12]. Nanocompounds, because of the large surface area, are implemented, increasing the sensing capability and selectivity [18]. However, for active transistors the interaction with the ambient induces instabilities in the transistor operation leading to degradation of the device performance. For ZnO as semiconducting film, oxygen and water molecules have a strong influence on the charge carrier concentration [19,20,21]. Oxygen molecules are chemisorbed, and they trap free electrons from the ZnO surface [O_2_ (g) + e^−^ → O_2_^−^ (ad)] reducing the free charge carrier concentration in the film and depleting its surface. Morrison reported that depending on the morphology of the semiconducting film, this surface effect can influence the whole film bulk [22]. Moreover, water molecules were also reported to partially desorb the oxygen molecules trapped at the ZnO surface increasing the film carrier concentration.

**Figure 2:**
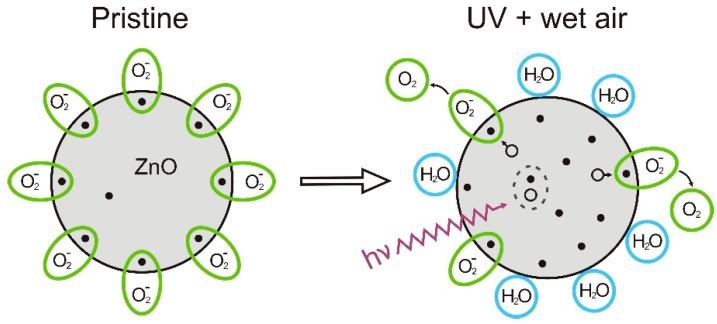
Schematic model of the stabilization of the ZnO nanoparticles.

Subsequent to the TFTs integration process, a high amount of oxygen is trapped at the ZnO nanoparticle surface. Due to their large surface area and the high amount of trapped oxygen the entire active semiconducting film is affected, thus the TFT shows very poor electrical characteristics. By storing the samples in a high humidity ambient (>50% relative humidity), the adsorbed oxygen is partially desorbed increasing the semiconducting properties of the film. The water molecules replace the released oxygen from the nanoparticle surface, increasing the number of free electrons in the film and the TFT’s drain current. Conversely, as this process saturates the entirely amount of oxygen trapped at the nanoparticles cannot be desorbed. This effect is mainly related to the difference in the ratio of chemisorbed and physisorbed oxygen, as well as in the defect density observed at the nanoparticle surface. In order to desorb a higher amount of trapped oxygen an UV irradiation step can be performed [20]. Electron–hole pairs are generated upon the illumination, and, due to the migration of holes to the nanoparticle surface, the chemisorbed oxygen is desorbed from the semiconductor. This process increases the charge carrier concentration in the semiconducting film and enhances the TFT performance. However, subsequent to the UV irradiation the highly reactive surface area induces a hysteretic behavior in the transistor transfer characteristic if the gate voltage is swept forward and backwards [23,24]. The instability is related to the dynamic adsorption and desorption of oxygen and water molecules by the application of an electric field during the operation of the transistor. This highly active surface can be stabilized by the adsorption of water molecules directly after the irradiation step, preventing the re-adsorption of oxygen molecules. Figure 2 depicts a schematic model of the interaction between the ZnO nanoparticle and the oxygen/water molecules during the stabilization process of the active semiconducting layer. The pristine ZnO nanoparticles are saturated with oxygen molecules, and a low charge carrier concentration is available for the current transport. As the UV irradiation matches the ZnO bandgap, electron–holes pairs are generated. The oxygen molecules are desorbed by the migration of holes to the nanoparticle surface. Subsequently, water molecules stabilize the highly active surface of the semiconductor.

After the stabilization of the ZnO nanoparticle film, the TFT electrical characteristics are stable and adequate for electronic circuit applications. A more detailed discussion and analyses of the TFT characteristics regarding the stabilization of the nanoparticles can be found in our previous work [23]. Furthermore, to prevent an overheating of the template, the UV irradiation is performed in 10 steps of 30 s of exposure and 30 s of recovery. During the pauses, the nanoparticulated ZnO can already adsorb oxygen inducting to an incomplete stabilization of the film. In order to avoid the re-adsorption of oxygen this treatment can be done in an atmosphere with reduced oxygen amount, which has been evaluated and discussed elsewhere [25]. The reduction of the oxygen amount and the purge of the released oxygen from the nanoparticles were done using a constant flow of nitrogen during the stabilization process. Conversely, the TFT instability increases due to the reduced amount of ozone present during the UV irradiation step. Ozone is reported to occupy the defects originated from oxygen vacancies at the nanoparticle surface increasing the connectivity between the nanoparticles [26,27].

### 2.2. Transistor Electrical Characteristics

**Figure 3:**
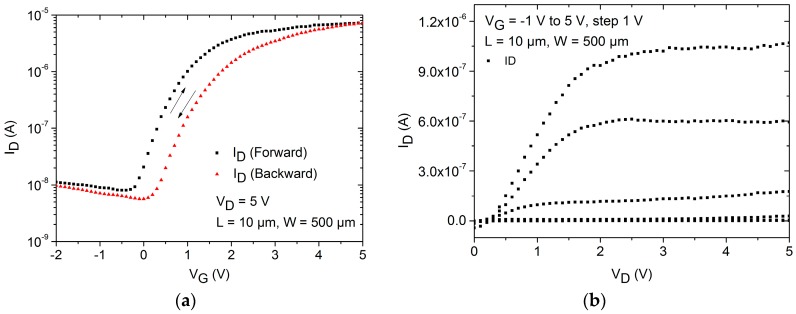
(**a**) Transfer and (**b**) output characteristics of a ZnO nanoparticle TFT applying an inverted coplanar setup.

Based on the reduced chemical and physical stress suffered by the active semiconducting layer, inverted coplanar structures are commonly used to evaluate the electrical characteristics of the semiconductor. Therefore, a spin-coating process was applied to deposit the water based ZnO nanoparticle dispersion followed by a solvent evaporation step. After the stabilization of the nanoparticle film by UV irradiation combined with wet-air, the transistors were electrically characterized. The transistor’s transfer and output characteristics are presented in Figure 3. It is possible to observe a small hysteretic behavior in the transfer characteristic when the gate voltage is swept forward and backward. This instability is related either to an incomplete stabilization of the nanoparticulated film or to the already existing defects at the dielectric/semiconductor interface. Additionally, as low temperature processes (maximum temperature of 115 °C) are used, no sintering process of the nanoparticles can be observed. The sintering of ZnO nanoparticulated films starts at temperatures above 400 °C and is reported to improve the nanoparticle interconnections [28]. Unfortunately, higher temperature processes are not applicable to polymeric substrates unavailing such approaches. Another approach for a punctual annealing of the nanoparticulated film is the employment of pulsed laser exposure. With this technique, the characteristic of the semiconductor can be optimized controlling the parameter of the laser processing or the annealing ambient [29,30]. Even though the annealing of the film is superficial, damaging on the semiconductor film [30] or gate dielectric by excessive laser exposure may lead to instabilities in the transistor operation. The TFT turn-on voltage (*V*_ON_) as defined by [14,31] is about 0 V and characterizes the switching point of the transistors. The *V*_ON_ is extracted from the log *I*_D_–*V*_G_ plot of the transfer characteristic at the point in which the drain current starts to increase from the transistor’s off state. The transistor off current is limited by leakage currents and by measurement system noises. Moreover, the *V*_ON_ is reported to avoid ambiguity originated from the non-idealities on the modeling of TFTs [14,31], and for this reason it was used in this study instead of the threshold voltage commonly used for the MOSFET characterization. A field-effect mobility of about 0.2 cm^2^·V^−1^·s^−1^ was extracted from the transistor transconductance, and the transistor depicts an *I*_ON_/*I*_OFF_ ratio in the range of 10^4^.

**Figure 4:**
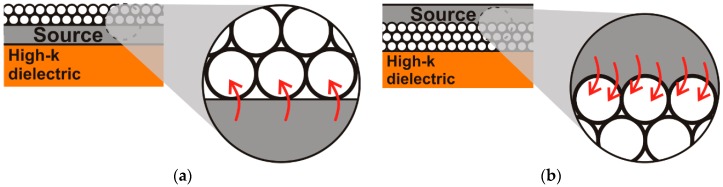
Detailed of the contact between the source (drain) electrode and the nanoparticulated layer in (**a**) inverted coplanar and (**b**) inverted staggered setups showing the charge carrier injection.

Due to the roughness between the drain/source electrodes and the nanoparticulated film, the transistor current is limited by the reduced contact area between both materials as well as by the low charge carrier injection at the contacts. Figure 4a shows in detail the contact and the charge carrier injection in inverted coplanar setups. 

Another disadvantage of the inverted coplanar setup is the critical definition of the spin-coating parameters during the semiconductor deposition. As the drain and source electrodes are already structured, the spin-coating process induces an uneven distribution of the semiconductor in the channel region due to the involved centrifugal forces. The decrease of the solid contents in the dispersion indeed improves the deposition uniformity on the wafer, but also reduces the contact quality between neighboring nanoparticles inducing a drastically increase of the semiconducting film resistivity. Therefore, the ZnO deposition can be improved by using a spray-coating technique. Besides the better compatibility for a later large-scale production on flexible substrates, this deposition method increases the yield of working transistors as well as the transistor performance. Previous works investigated the deposition using spray coating technique on freestanding polymeric substrates [17]. Despite the hindrances concerning the integration on flexible templates, these transistors depict an improved performance in comparison to the ones using spin-on semiconducting layers. The field-effect mobility is reported to be about 0.5 cm^2^·V^−1^·s^−1^, the *I*_ON_/*I*_OFF_ ratio about 10^5^ and the *V*_ON_ about 1 V. Even applying different deposition methods for the active semiconductor and using either an oxidized Si wafer or a polymeric substrate, the transistor characteristics present no significant variation. This constancy depicts the robustness of the developed integration process.

The main drawback of inverted coplanar setup is the poor contact quality between the drain and source materials and the semiconducting film. Hence, by applying an inverted staggered setup, the drain and source electrodes are structured on top of the semiconducting layer and a better contact quality can be achieved. In this case, the gaps between the nanoparticles are filled by the drain/source material increasing the contact surface between both materials. Conjointly, an improved charge carrier injection through the contact is also expected. Nevertheless, staggered setups have the disadvantage that the drain and source electrodes are not in direct contact with the formed accumulation channel, and the charge carrier have to cross the semiconducting layer thickness to reach this conductive channel. Conversely, this effect is reported to be negligible in comparison to advantages of the improved contact area between the drain/source and the semiconductor [32]. Additionally, as the deposited semiconductor commonly presents unconformities as valleys, peaks and pin holes, the charge carrier path to reach the channel is reduced. The enhanced contact area for inverted staggered setup can be observed in the Figure 4b.

**Figure 5:**
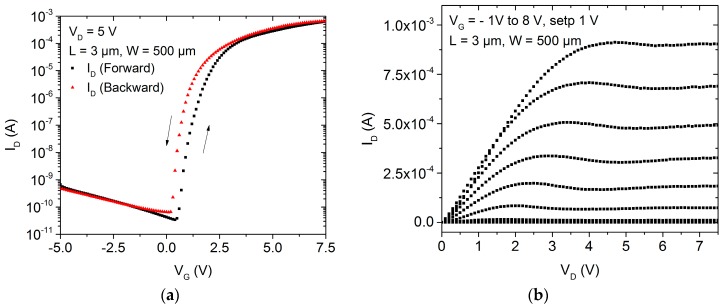
(**a**) Transfer and (**b**) output characteristics of a ZnO nanoparticle TFT applying an inverted staggered setup.

The expected performance improvement when the inverted staggered setup is applied can be noted by the transistor transfer and output characteristics shown in Figure 5. The field-effect mobility has increased from about 0.2 cm^2^·V^−1^·s^−1^ to 3.7 cm^2^·V^−1^·s^−1^ and the *I*_ON_/*I*o_FF_ from 10^4^ to 10^7^. The turn-on voltage is about 0.5 V and has not significantly shifted in comparison to the inverted coplanar setup either using spin-coating or spray coating deposition techniques. From the *I*–*V* curve of the transistor it is possible to note that the on-state current level is about 100 times higher. This current level is attributed to the better charge carrier transport through the drain and source contacts.

The improved contact quality is ascribed to the increased contact surface between the drain and source electrodes and the nanoparticulated film. A precise and reliable estimation of the contact resistance value and the mechanism responsible for the charge carrier injection, however, is still under investigation. The hindrances are related to the semiconducting film morphology. As the ZnO nanoparticles creates percolation paths for the current transport, this characteristic induces to discrepancies in the estimation of the resistance value. Additionally, as the metal–semiconductor contacts are influenced by the applied bias, a non-linear and non-ideal behavior is also commonly observed [33]. Studies reporting on the conduction mechanisms in non-crystalline ZnO films can be found elsewhere [34,35]. The current transport is strongly affected by the density of defects and the film grain boundaries. When using ZnO nanowires, for instance, the orientation of the nanowires and network formation are even more critical, as a poor electrostatic coupling between the semiconducting material and the gate electrode is observed requiring higher gate voltages for the device operation [36,37]. 

To the best of our knowledge, the transistor metrics observed for the inverted staggered setup are among the highest electrical performances reported for ZnO nanoparticle TFTs. In comparison with the results presented in this study, Faber et al. have described TFTs depicting higher charge carrier mobilities, however they show a highly pronounced hysteresis in the transfer characteristic. For an improvement of the performance, the ZnO nanoparticles were treated in oxygen plasma [38]. This approach induces instabilities on the transistor operation and prevents the integration on flexible substrates as the oxygen plasma damages the substrate as well as polymeric dielectrics. Cho et al. have reported ZnO based TFTs integrated using nanoparticles and precursor mixture ink in order to achieve a better film morphology enhancing the transistor performance [39]. However, for the formation of the semiconducting film temperatures of 250 °C are required limiting the compatibility of the integration process to certain polymeric substrates or glass. Park et al. have also discussed TFTs with similar characteristics applying alkali metal doped ZnO as active semiconductor; nevertheless, in this case, higher temperature processes are also necessary [40]. The electrical characteristics are also comparable to the ones of TFTs with semiconductors deposited using ZnO precursors, however high annealing temperatures or annealing under strict atmosphere are essential to reduce instabilities and to assure the operation at low voltages [41,42]. ZnO based TFTs using sputtering techniques depict denser semiconducting films besides the possibility to vary the semiconductor composition by adding In, Ga or Sn, for example. These TFTs commonly present higher performance [43,44,45], notwithstanding the transistors reported in this study present similar metrics using low-cost fabrication processes and materials as well as an integration process that is fully compatible to flexible substrates. 

### 2.3. Inverter Characteristics

**Figure 6:**
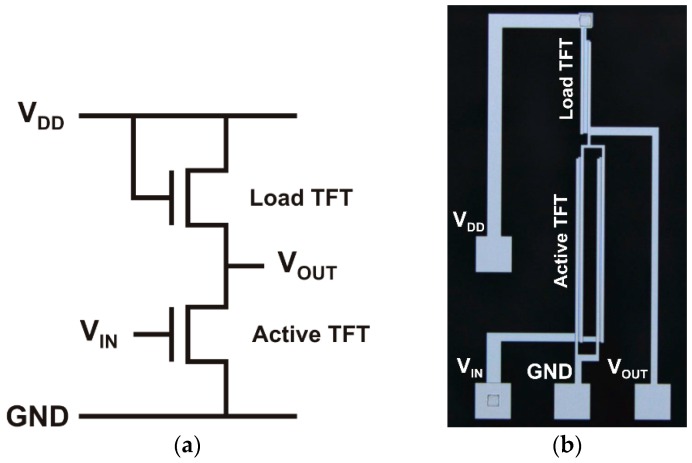
(**a**) Schematic circuit and (**b**) an optical microscope image of an inverter using ZnO nanoparticle TFTs.

Since the inverted staggered setup for the ZnO nanoparticle TFT has presented an improved performance, inverter circuits were also integrated to evaluate their characteristics for digital circuit applications. The inverters were fabricated using a load transistor in the pull-up network and an active transistor in the pull-down network. Figure 6 shows the schematic circuit and an optical microscope image of an inverter.

**Figure 7:**
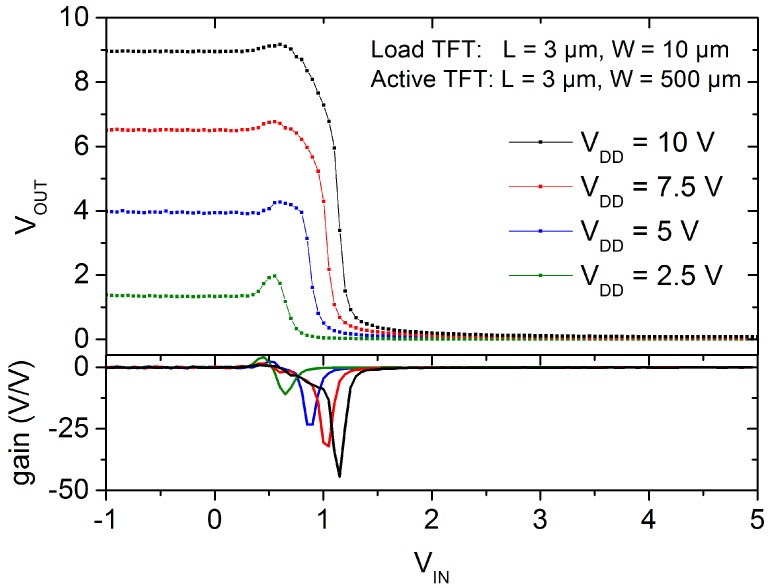
Voltage transfer characteristic of a ZnO nanoparticle inverter with different supply voltages. The bottom graph depicts the gain in dependence on the input voltage.

The voltage transfer characteristic (VTC) of a typical inverter with different supply voltages (*V*_DD_) is depicted in Figure 7. The inverter circuit shows *V*/*V* peak gains of about 11 for *V*_DD_ = 2.5 V and of around 45 for *V*_DD_ = 10 V. Additionally, the high and the low output voltage levels swing almost the entire supply voltage course, i.e., the high output level is comparable to the *V*_DD_ and the low output level is close to the ground potential; although the inverter uses a single type of transistor (load and active TFTs) instead of a complementary design. Another important aspect of the inverter is the noise margin defining the voltage tolerance or amount of noise that the circuit withstands without compromising its operation. This margin is defined for low levels as *NM*_L_ = *V*_IL_ − *V*_OL_ and for high levels as *NM*_H_ = *V*_OH_ − *V*_IH_. The input low voltage (*V*_IL_), the output low voltage (*V*_OL_), the output high voltage (*V*_OH_) as well as the input high voltage (*V*_IH_) are extracted from the voltage transfer characteristic at the points where the gain *V*/*V* of the inverter is equal to the unit. For the inverter characterized in Figure 7, the noise margin are about *NM*_L_ = 0.4 V, independently of the supply voltage, and the *NM*_H_ is about 1 V for *V*_DD_ = 2.5 V and around 7.6 V for *V*_DD_ = 10 V.

**Figure 8:**
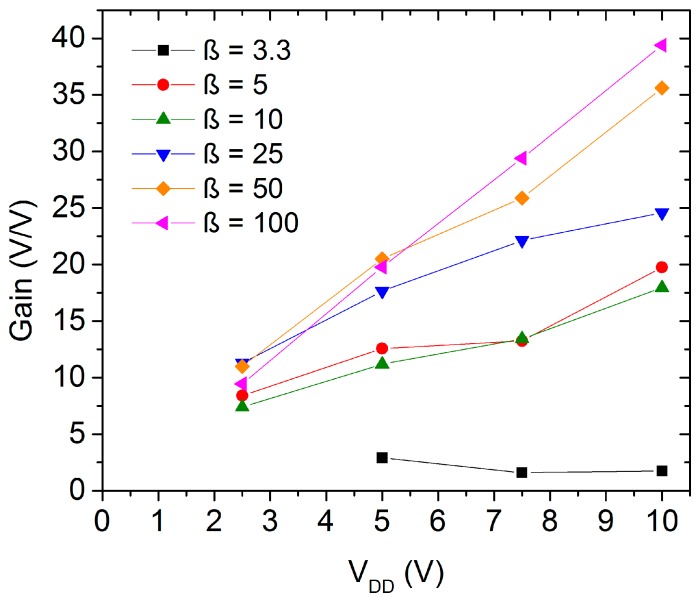
Average gain of the ZnO nanoparticles inverters in dependence on the supply voltage.

**Figure 9:**
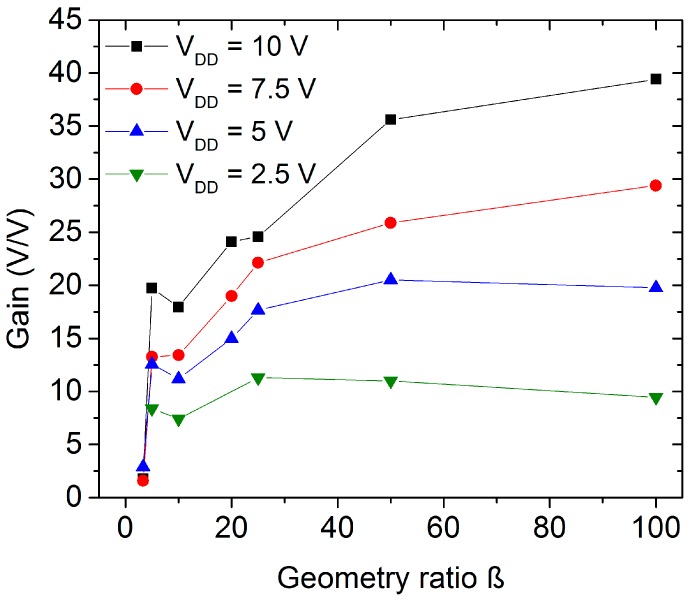
Average gain of the ZnO nanoparticles inverters in dependence on the geometry ratio.

The inverter geometry, especially the difference between the load and active transistors’ sizes, affects the circuit characteristics, for instance the gain and the power consumption. The geometry ratio (β) is defined as the quotient between the *W*/*L* relation of the active and the load transistor. Figure 8 depicts the average peak gain of the inverter structures with different supply voltages and geometry ratios. An increase of the peak gain is observed when a higher supply voltage is applied. This effect was noted in all analyzed inverter geometric ratios but β = 3. In this case, the active TFT requires a large variation of the input voltage to switch the output voltage level decreasing the inverter gain. Figure 9 shows the peak gain as a function of the inverter geometry. On the one hand, it is possible to recognize an improvement on the inverter gain following the increase in the size difference between the active and the load TFT. On the other hand, the inverter circuits gain starts to saturate at β > 25 indicating that a higher geometry ratio consumes more active area of the substrate without a substantial improvement of the inverter performance increasing the production cost.

**Figure 10:**
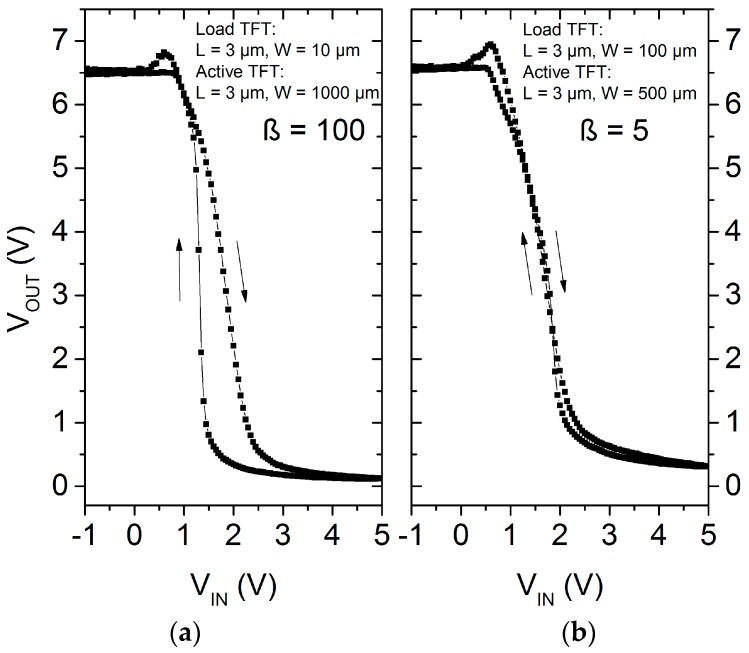
Voltage transfer characteristics of a ZnO nanoparticle inverter with geometry ratio (**a**) equal to 100 and (**b**) equal to 5 and with *V*_DD_ = 7.5 V.

The hysteretic behavior observed in the *I*–*V* curves of the transistors is also transferred to the electrical characteristics of the inverter. An inverter with β = 100 depicts a difference in the switching point of about 0.6 V when the input voltage (*V*_IN_) is swept from logic level 0 to 1 and again vice versa, as shown in Figure 10a. This shift in the operation characteristics is proportional to the variation of the *V*_on_ observed during the TFT characterization. Inverters with smaller geometric ratio present a lower shift difference (see Figure 10b) due to the operation point of the active TFT, which is less sensitive to the hysteretic behavior. However, the inverter requires a higher excursion of the *V*_in_ to switch the output level. An approach to improve the switching point and increase the inverter reliability could be done by, for instance, a more robust circuit design [46].

**Figure 11:**
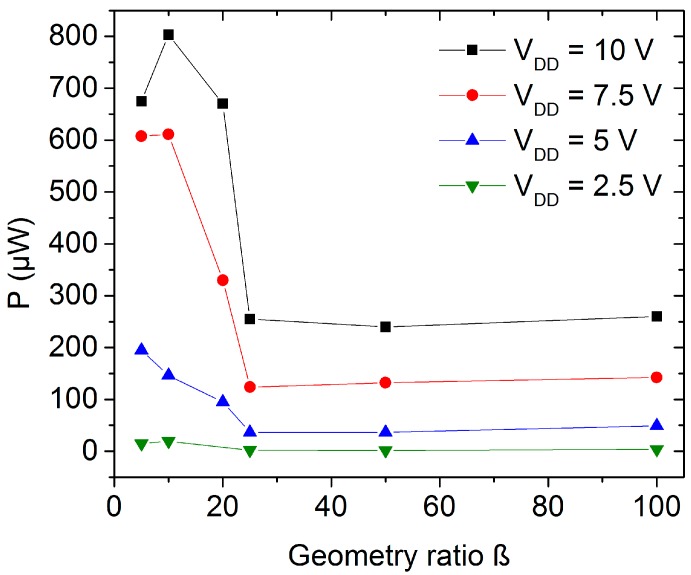
Power consumption as function of the ZnO nanoparticle inverter geometry ratio.

Commonly, inverter circuits applying a single TFT type with load and active transistors consume higher power. Figure 11 shows the power consumption as function of the inverter geometry ratio; for inverter with low β the consumption is higher as the pull-up transistor allows a relative high current flow through the circuit. The main consumption is when *V*_out_ is at low voltage state (*V*_in_ is at high voltage state) as both load and active TFTs are conducting. During the high voltage state of the output, the power consumption decreases to less than 0.1 µW for all geometry ratios as the active transistor is not conducting.

Further improvements regarding the power consumption and the switching characteristics of the inverters can be achieved by using a complementary design. Therefore, an organic based TFT presenting *p*-type TFT characteristics could be used in the pull-up network instead of a load TFT. Nevertheless, the performance of the inverters presented in this study shows adequate characteristics for the integration of digital circuits on flexible templates as the integration process is fully compatible to polymeric substrates. Moreover, the inverter metrics are comparable to devices that are integrated using sputtering techniques and high performance materials or high annealing temperature processes for the semiconducting layer [47,48].

## 3. Materials and Methods

**Figure 12:**
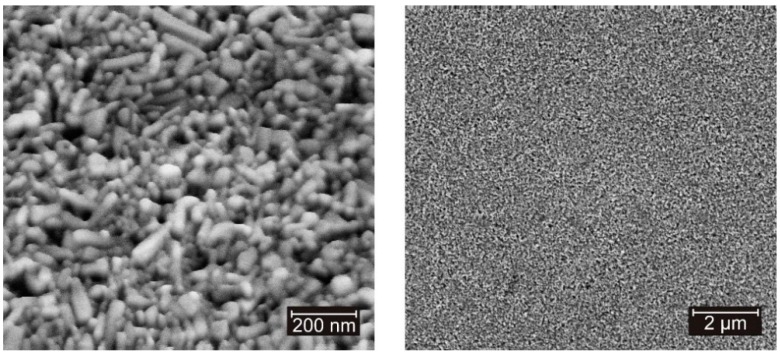
Scanning electron microscope images of the ZnO nanoparticle layer after the solvent evaporation at 115 °C in a convection oven for 1 h and the UV/wet-air treatment.

The ZnO nanoparticle based TFTs and inverters were integrated on oxidized silicon wafer using processes that are fully compatible to flexible substrates, as evaluated previously on polyethylene terephthalate (PET) substrate [17]. The silicon wafer was only used as mechanical support for the integrated devices and as a method to evaluate their performance prior to the transfer of the process to a polymeric template. For the inverted coplanar structures, a layer sequence of 50 nm aluminum and 7 nm titanium was e-beam evaporated. The gate electrodes were formed by a contact photolithography technique followed by wet etching processes. A spin-on high-*k* resin (*k* = 12 [49]) was used as gate dielectric. This material purchased from Inomat GmbH (Neunkirchen, Germany) is based on hydrolyzed and partial condensed ethyl silicates filled with TiO_2_ nanoparticles (trade name: Inoflex [50]). This organic-inorganic nanocomposite combines the advantages of the polymeric matrix (e.g., flexibility) and of the inorganic compound (e.g., high dielectric constant). After its deposition, the dielectric resin was cured and cross linked by a thermal treatment at 115 °C for 30 min in a convection oven in ambient atmosphere and by an UV (λ = 365 nm and 200 W/cm^2^) irradiation step (4 min of irradiation done in steps of 40 s exposure and 60 s of pause to prevent excessive substrate heat). A final dielectric thickness of about 150–180 nm was achieved. To contact the gate electrode, via contacts were opened through the gate dielectric using photolithography and wet chemistry techniques. Subsequently, a 150-nm-thick aluminum layer was evaporated under high vacuum conditions and structured by photolithography and wet-chemistry processes to form the drain and source electrodes. The water based ZnO nanoparticle dispersion purchased from Nanophase Technologies Corporation (Romeoville, IL, USA) with average particle size of around 70 nm [51] was deposited on the template by spin-coating technique. After a baking process at 115 °C for 1 h in a convection oven in ambient atmosphere, the nanoparticulated layer was stabilized by an UV irradiation step (5 min of irradiation done in steps of 30 s exposure and 30 s of pause). Directly after this step, the template was stored in a high humidity (RH > 50%) chamber for 30 min. Figure 12 shows scanning electron microscope images of the nanoparticulated layer after the UV/wet-air treatment. A semiconducting film of about 300 nm thick was achieved; nevertheless, the thickness is influenced by the topology of structures underneath. 

For the inverted staggered setup, the processes up to the gate dielectric deposition and curing step are the same as the ones followed for the inverted coplanar setup. After a chemical activation of the dielectric layer surface, the nanoparticle dispersion was deposited by spin-coating technique, cured and treated using UV irradiation combined with wet-air, as previously described for the inverted coplanar setup. Via connections through the semiconducting layer and the gate dielectric were opened to contact the gate electrode. The drain and source electrodes as well as the TFT connections to form the inverter circuits were structured by lift-off technique of a 150-nm-thick aluminum layer evaporated under high vacuum conditions. After the integration process, the devices were characterized in a dark environment at room temperature under ambient atmosphere using a HP 4156A Precision Parameter Analyzer (Santa Rosa, CA, USA).

## 4. Conclusions

In summary, aiming at the integration of cost efficient TFTs for flexible and transparent electronics, we have discussed the electrical characteristics of ZnO TFTs applying inverted coplanar and inverted staggered setups. Because of the improved contact quality between the drain and source electrodes and the ZnO nanoparticle layer, a higher charge carrier injection is achieved increasing the transistor performance of inverted staggered structures. The TFT metrics are among the highest reported for nanoparticulated based devices and are comparable to TFT, which uses high cost processes or expensive metal oxides compound, as well as high temperature annealing steps. 

In order to evaluate the transistor characteristics in digital circuit applications, inverter circuits were integrated. The devices were characterized evaluating the dependence on the inverter geometric ratio and the supply voltage. The inverters depict high *V*/*V* gains and adequate switching point characteristics. Future works will be focused on the transfer of the integration process to polymeric substrates and on the dynamic characterization of the inverters using ring oscillator circuits. Additionally, one part of the current efforts is the development of a complementary design using DNTT and C_8_-BTBT based TFT in the pull-up network of the inverter to improve the switching characteristics as well as the power consumption.

## Abbreviations

The following abbreviations are used in this manuscript:

C_8_-BTBT	2,7-dioctyl[1]benzothieno[3,2-*b*]benzothiophene
DNTT	Dinaphtho[2,3-*b*:2′,3′-*f*]thieno[3,2-*b*]thiophene
*NM*_H_	Noise margin for high levels
*NM*_L_	Noise margin for low levels
MOSFET	Metal-oxide-semiconductor field-effect transistor
RH	Relative humidity
TFT	Thin-film transistor
UV	Ultra-violet
*V*_IH_	Inverter circuit input high voltage
*V*_IL_	Inverter circuit input low voltage
*V*_OH_	Inverter circuit output high voltage
*V*_OL_	Inverter circuit output low voltage
*V*_ON_	Turn-on voltage
VTC	Voltage transfer characteristic
